# Wear Distribution Detection of Knee Joint Prostheses by Means of 3D Optical Scanners

**DOI:** 10.3390/ma10040364

**Published:** 2017-03-30

**Authors:** Saverio Affatato, Maria Cristina Valigi, Silvia Logozzo

**Affiliations:** 1 Medical Technology Laboratory, Rizzoli Orthopaedic Institute, 40136 Bologna, Italy; 2 Department of Engineering, University of Perugia, 06125 Perugia, Italy; mariacristina.valigi@unipg.it (M.C.V.); silvia.logozzo@vger.eu (S.L.); 3 Department of Research, V-GER S.r.l., Via Mori 6, Prunaro di Budrio, 40121 Bologna, Italy

**Keywords:** 3D wear map, knee joint, prostheses design, 3D scanners, wear inspection

## Abstract

The objective of this study was to examine total knee polyethylene inserts from in vitro simulation to evaluate and display—using a 3D optical scanner—wear patterns and wear rates of inserts exposed to wear by means of simulators. Various sets of tibial inserts have been reconstructed by using optical scanners. With this in mind, the wear behavior of fixed and mobile bearing polyethylene knee configurations was investigated using a knee wear joint simulator. After the completion of the wear test, the polyethylene menisci were analyzed by an innovative 3D optical scanners in order to evaluate the 3D wear distribution on the prosthesis surface. This study implemented a new procedure for evaluating polyethylene bearings of joint prostheses obtained after in vitro wear tests and the proposed new approach allowed quantification of the contact zone on the geometry of total knee prostheses. The results of the present study showed that mobile TKPs (total knee prosthesis) have lower wear resistance with respect to fixed TKPs.

## 1. Introduction

To evaluate new total knee prosthesis (TKP) designs, over the last 15 years or so, strong efforts have been made to improve the design, the material properties, and the method to enhance the wear measurements [1,2]. This has also partly been a response to the increase in regulatory and pre-clinical testing required for assessing product reliability and patient safety. Current TKP devices can be subdivided into two groups based on different rotational degree of freedom along the tibial axis for the ultra-high-molecular-weight-polyethylene (UHMWPE) insert. In particular, a fixed design means that the UHMWPE insert is locked with the tibial tray; conversely, the mobile designs facilitate the movement of the insert relative to the tray [3].

Upon simulation of clinically relevant wear, it is then important to be able to accurately quantify this wear and the consequent TKP functionality. Various approaches have become standardized and currently, in in vitro research studies, the most commonly used measurement method for wear assessment is the gravimetric method [4]. By operating with this method, a microbalance is used to measure the mass loss of the specimens during a wear test. The weight loss is then calculated as the difference of the two measurements. On one side, this method has proven to be sufficiently accurate to globally quantify the worn material, on the other side it cannot detect dimensional changes. Wear volume measurement and geometrical characterization of worn bearing surfaces are fundamental for understanding wear mechanisms and improving technological solutions provided by manufacturers of prosthetic joint components [5,6,7].

In assessing the applicability of new wear measurement methodologies, currently, the coordinate measuring machine (CMM) remains the most obvious method for measuring the geometry of joint prostheses [8,9]. Some authors assessed the wear of commercial ultra-high-molecular-polyethylene (UHMWPE) acetabular cups by using a micro X-ray computed tomography technique as an alternative to overcoming the limitations of the gravimetric method. In particular, Teeter M.G. et al. [10,11,12,13] validated the micro-CT technique to measure surface deviation and therefore calculate the volume of wear plus creep of retrieved acetabular liners. Accordingly, Engh et al. [14] used a micro-CT technique to quantify the polyethylene wear on retrieved knee components and they found that the micro-CT technique can determine the volume and location of the wear of retrieved tibial liners. Recently, Parrilli and co-workers [15] used a micro-CT technique to measure the volume loss from ceramic femoral heads. Affatato et al. [16] validated a micro X-ray computed tomography technique to assess the wear of different commercially available, polyethylene’s acetabular cups after wear simulation. Real-time wear was also measured by ultrasonic reflectometry and interferometry. Kurtz et al. [17] studied the volumetric wear for ultra-high molecular weight polyethylene (UHMWPE) joint replacement components. They quantified the wear rate on acetabular components by white light interferometry. Saarakkala et al. [18] employed quantitative ultrasound imaging to detect degenerative changes in articular cartilage surface and subchondral bone. Passos et al. [19] employed the method of focus variation microscopy (FVM) for the analysis of human enamel and dentin subjected to erosion/abrasion.

Austin et al. [20] used the technique of confocal laser scanning microscopy and area-scale analysis to quantify enamel surface textural changes from citric acid demineralization and salivary remineralization in in vitro experiments.

With this in mind, a new wear assessment approach was proposed in order to quantify complicated geometry, such as the menisci of a TKP. Wear and deformations of a TKP were then assessed by means of advanced 3D optical scanning techniques [21,22]. In particular, wear distribution and deformations depend on the load configuration and positioning adopted during the wear progress; therefore, with this new approach, we can assess both the volume wear rates and the 3D wear distribution over the specimen’s surface, even if the measured object has a complex shape. Furthermore, no contact probes are needed to perform the measurement, as measurement is performed by CMMs, and this represents an advantage as it allows the roughness and morphology of the measured surface to be preserved. With respect to the weighing method, it is useful to assess the amount of the worn volume only when the exact density of the material is known and when the material is supposed to be homogenous.

The aim of the present study is to assess the feasibility of using advanced 3D optical scanning techniques to experimentally establish the areas mostly affected by wear in order to gather basic information to allow a better design of prosthetic knee joints in the future.

## 2. Materials and Methods

**Figure 1:**
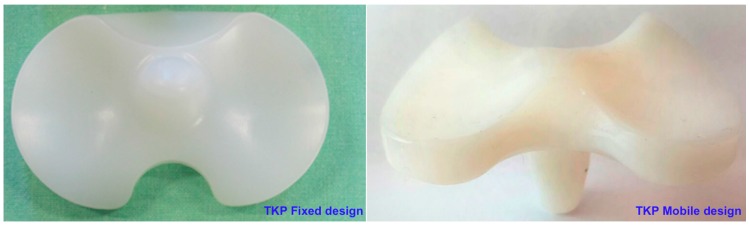
Total knee prosthesis (TKP) fixed and mobile design used in this study.

The wear behavior of commercially available TKP fixed and mobile bearing configurations (four specimens for each batch, Adler ORTHO, Milan, Italy, Size #2) was investigated. Each polyethylene tibial insert (meniscus) was coupled with CoCrMo femoral components. A picture of the mobile and fixed menisci design is shown in Figure 1.

The conventional polyethylene resin (GUR 1050) was surgical grade, consolidated by compression molding (according to ISO 5834/2) and Ethylene Oxide (ETO) sterilized. Following a standardized protocol, all the UHMWPE tibial components were pre-soaked for four weeks prior to the wear tests in order to achieve a steady level of fluid absorption, as recommended by the international standard (ISO 14243-2) [23]. After this procedure, three of these UHMWPE tibial components were put in run onto the simulator (in dynamic mode) and the last one was used as the check control (the two configurations were considered fixed and mobile).

### 2.1. Wear Test Details

The wear test was performed using a “three-plus-one” stations displacement-controlled simulator (Shore Western Mfg., Monrovia, CA, USA). The knee simulator is the same machine as used for other knee wear tests: in particular, three stations were used for the test specimens and the last one was used as the soak control specimen in order to estimate the total change in mass due to lubricant absorption, as recommended by the ISO 14243-2. The lubricant used was 25% (vol/vol) sterile bovine calf serum (Euroclone, Milano, Italy) balanced with deionized water and 0.2% sodium azide (Sigma Chemical, St. Louis, MO, USA) to slow down bacterial degradation. Ethylenediamine-tetra-acetic-acid (20 mmol/dm^3^) was added to minimize the precipitation of calcium phosphates on the bearing surfaces, which strongly affects friction and wear properties. The applied kinematics was in displacement control and all the movements (axial load, anterior/posterior translation, intra/extra-rotation, flexion/extension) replicated a simplified gait cycle according to ISO 14243-1/3. Load was applied vertically (perpendicular to the tibial tray and oscillated in the range 168–2600 N. Major details are available in previous work [24].

### 2.2. Specimens Preparation before Wear Inspection

All the UHMWPE menisci were vacuum washed before acquiring the wear map. In particular, considering that the polyethylene resulted to be semi-transparent (light passed through the components and it was hard to acquire by active optical 3D scanning methods) and in order to achieve optimal accuracy and results with 3D optical scanning, all the specimens were matted by applying a special anti-glare coating powder (Laser scanning anti-glare spray, Helling GMBH, Heidgraben, DE, Germany). This powder provides a uniform coverage and thanks to the fine-grained structure of the spray, it is possible to evenly apply layers of spray with minimal thickness (average particle size of 2.8 µm) [25]. The uniform coverage was assured by spraying the prosthesis in a painting booth with controlled conditions. 

Moreover, all the specimens were weighed with and without coatings before scanning using a microbalance (Sartorius Cubis MSE 225 S-000-DU, Goettingën, Germany) with a precision of 0.01 mg. The weights of the matted prostheses were used to check that the weight of the powder coating was almost the same for every specimen with a difference of ±2 mg.

### 2.3. 3D Optical Scanning Procedure

**Figure 2:**
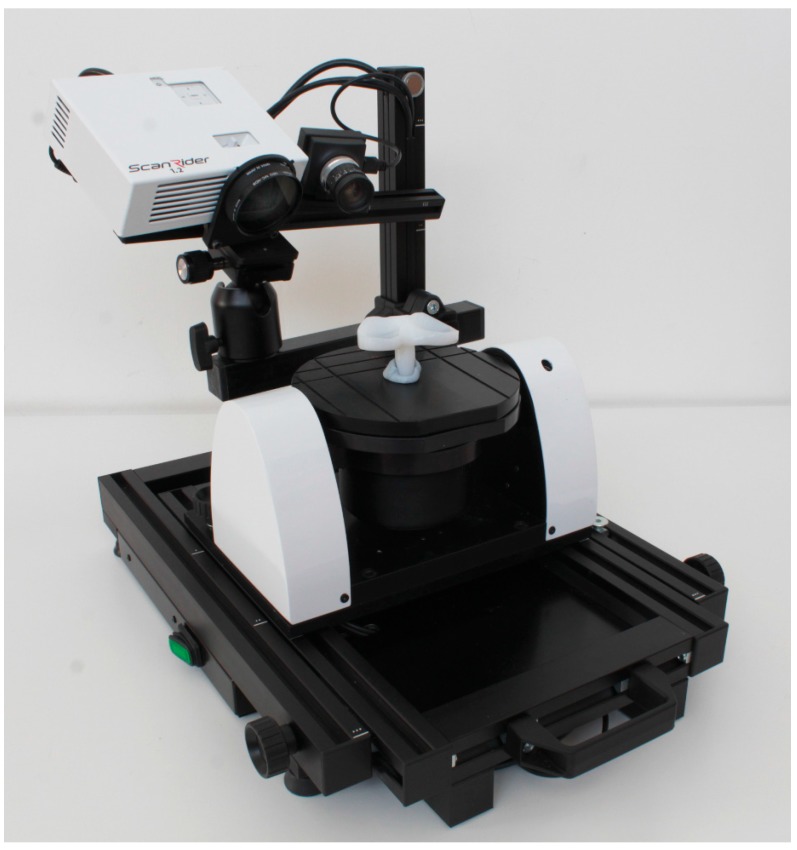
ScanRider 3D, automated metrological optical scanner while scanning a mobile meniscus.

The metrological device used in this work is a 3D scanner (ScanRider, model 1.2, V-GER SRL, Bologna, Italy) based on the principle of structured light active triangulation (Figure 2).

This instrument, used in previous works [26], is a completely modular 3D optical scanner, allowing automatic scanning procedures and providing the full 3D digital model of the specimen without the intervention of the operator, thanks to the automated positioning worktable. The device can be adjusted to scan specimens of a wide range of sizes and avoid the use of physical markers. In this device, the light source is a 600 lumen white, blue, red or green light projector, projecting light stripe-structured patterns on the scene. The different projectable light color allows the exploitation of the natural optical characteristics of the different surfaces and limits noise. The highly accurate optical calibration system allows to assess the real precision of the instrument at each measurement. The technical specifications of the instrument assure a resolution better than 0.05 mm, a precision better than 0.03 mm and an accuracy better than 0.01 mm for a scanning volume of 60 × 50 × 50 mm, as shown in Table 1.

Each specimen (sample and check prosthesis) was scanned by performing automatic cycles: eight rotating positions and three tilting positions in order to have 24 different scanned perspectives. Each prosthesis was measured three times and the 3D digital models were compared to obtain information about the deviation. An average volume deviation of 0.01% was found that is negligible for the purpose of this study, as is clear by looking at the quantitative results in the following paragraphs. In order to optimize the mesh quality (taking care to preserve the original shape and volume of the 3D data), the full 3D digital model obtained by the above scanning procedure was processed by using the mesh editing software VXmodel (Creaform Inc., Lévis, QC, Canada). The steps for the 3D data optimization included the detection and restoration of edges, self-intersections, highly creased edges, spikes, small components and holes. The average entire mesh size was set around 1.000.000 triangles and the number of triangles was kept similar in each sample meniscus and its check, in order to obtain comparable results. After the data optimization, the 3D models of the sample prostheses (worn models) were compared with the corresponding reference nominal 3D models, represented by the digital models of the check prostheses. In this case, as the tolerances of the production process are not negligible with respect to the order of magnitude of the wear, the computer aid design (CAD) models were not considered efficient for the comparison.

**Figure 3:**
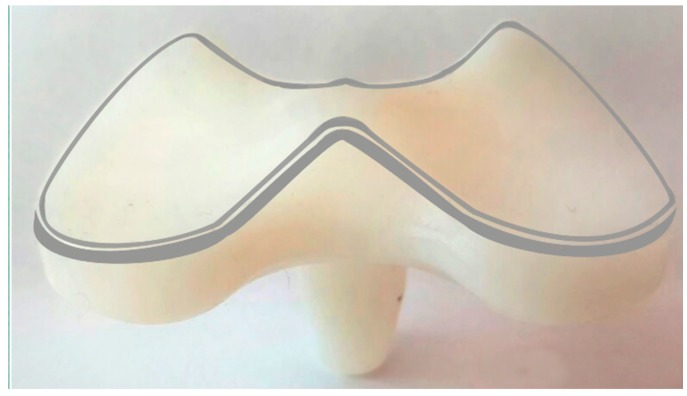
Unworn portions (grey areas) taken into account for the alignment.

The software used for the digital inspection process was VXinspect by Creaform (Creaform Inc., Lévis, QC, Canada). The digital inspection procedure was performed by:

- Evaluating deviations by aligning and superimposing the worn (sample) and unworn (check) models. The alignment was performed by taking the unworn portions of the contact area of the menisci as a reference. Those unworn parts are localized especially at the edges of the prosthesis, as shown in Figure 3 (grey areas). The superimposition was performed by fine registration algorithms, based on best-fit criteria and iterative closest point (ICP) algorithms.
- The 3D color wear map was created considering the contact part of the menisci, as it is the part mainly affected by wear.

## 3. Results

### 3.1. Gravimetric Wear Results

**Figure 4:**
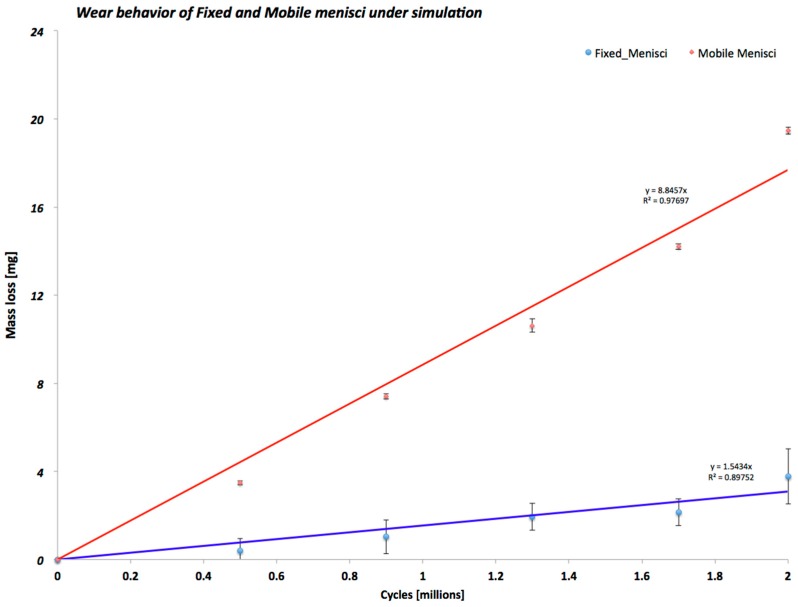
Wear trend and linear regression of the different fixed and mobile TKP configurations during the wear test.

As shown in Figure 4, during all wear tests, the mobile menisci configuration had almost ten times as much wear as the fixed menisci configuration. The wear behavior and regression coefficient for the different TKP configurations tested emphasize that the fixed menisci configuration maintained a constant wear behavior until the end of the test. On the contrary, the mobile menisci configuration demonstrated an increased mass from 0 to the end of the test.

### 3.2. 3D Wear Inspection Results

In order to characterize the surface of the TKP configurations, 3D optical scanning measurements were carried out on all specimens (worn prostheses and checks) after the end of the wear test, in order to compare the difference in shape and volume of some parts around the contact area of the two studied TKP configurations. The comparison was performed by using two different input data sets: the 3D digital models of all the worn specimens and of all the checks. Two different comparison methods were used: in the first case, both for the fixed and mobile menisci, the entire worn prosthesis was considered and aligned with the corresponding check on the basis of the unworn regions (including the lateral surface, not involved in the contact); in the second case and only for the fixed menisci, we took into account only the contact region (so-called butterfly area), both on the worn sample and on its check. The consideration of this butterfly area allows misalignments—due to the different configurations of the pin on the mobile menisci surface—to be avoided.

**Figure 5:**
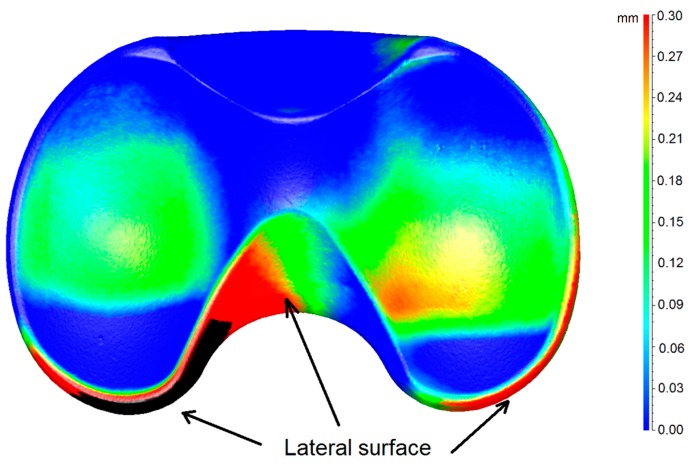
3D wear map of the TKP mobile meniscus #1.

In Figure 5, the 3D color wear map of the mobile meniscus #1 is shown, as this meniscus resulted to be the most worn of the entire group. The map is the result of the calculation of deviations after the superimposition of the 3D digital model of the worn sample and of its check. Thus, in the picture, both the meniscus #1 and its check are represented. In particular, the color map represents the 3D material loss distribution on the joint surface in mm, taking the worn model as a reference. 

From this picture, we can observe that there are little differences between the worn (meniscus #1) and the unworn (check) models around the lateral surface, which is not involved in the friction contact. This is due to the tolerance of the production process. In this case, this error does not affect the measurement results, as the reference and worn model were perfectly aligned, taking into account only the unworn boundaries of the region involved in the contact, where there is a perfect fit, as shown in the picture (blue color over all the peripheral regions).

**Figure 6:**
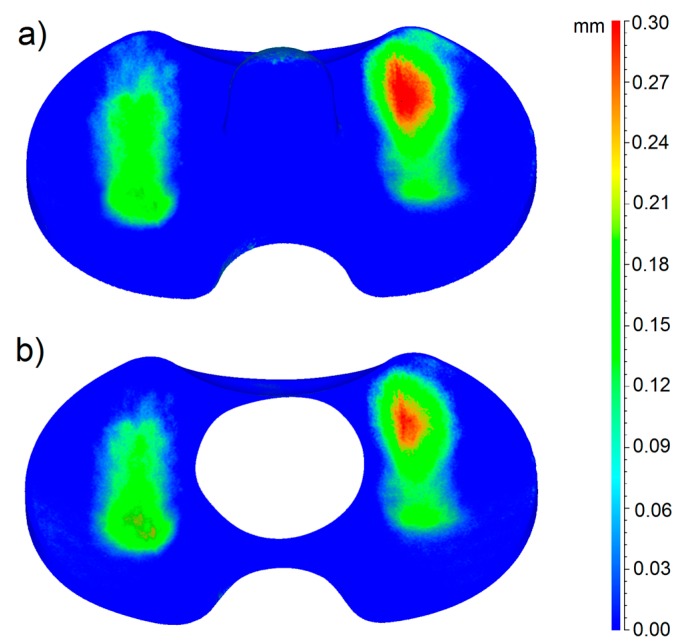
3D wear map of the fixed meniscus #4: in part **a**, the 3D wear map of the entire prosthesis is shown; in part **b**, the 3D wear map of the butterfly region is shown.

**Figure 7:**
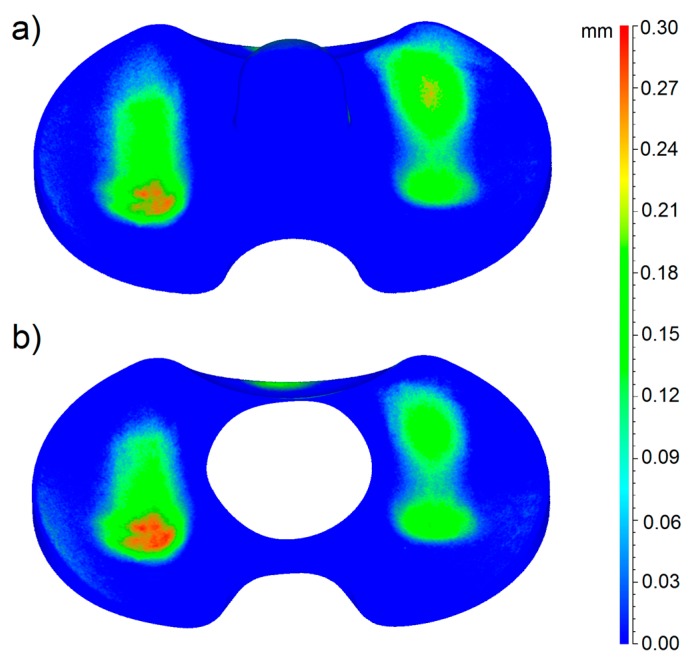
3D wear map of the fixed meniscus #2: in part **a**, the 3D wear map of the entire prosthesis is shown; in part **b**, the 3D wear map of the butterfly region is shown.

In the case of the mobile menisci, the butterfly area was not taken into account, as the correct alignment of the worn and unworn models is not affected by anything on the menisci surface. In Figure 6 and Figure 7, the 3D color wear maps of the fixed menisci #4 and #2 are shown. Also in this case, these menisci resulted to be the most worn of the entire group. The map is the result of the calculation of deviations after the superimposition of the 3D digital model of the worn sample and of its check, taking the worn model as a reference. Again, both the menisci #4 and #2 and their checks are represented in the pictures.

In particular, the blue color represents the areas not affected by wear and the red color indicates the areas most affected by loss of material. Figure 6 and Figure 7 also show that the menisci #4 and #2 and their checks belong to the same production batch; in fact, they present a perfect fit, especially on the contact area, but also on the peripheral and lateral regions. 

From the gravimetric tests and from the 3D models of the menisci, the mass and volumetric wear rates were evaluated and compared in order to validate the method: the deviation found is approximately 10%. In particular, Table 2 shows detailed results obtained by fixed menisci analysis.

The wear rates can be calculated according to the following equation:

(1) $$\chi =\;\frac{V_{i}-\;V_{f}}{V_{i}}\%=\;\frac{P_{i}-\;P_{f}}{P_{i}}\%.$$

where *V_i_* and *P_i_* are the volume and weight measured in the unworn model (check) and *V_f_* and *P_f_* are the volume and weight measured in the 3D model of the worn component. 

The mass wear rates were evaluated taking into account the weights measured after the application of the matting powder, in order to also consider the thickness and weight of the powder layer and to obtain results comparable with the volumetric results. Furthermore, the volumes were measured considering the butterfly area.

## 4. Discussion

Although surgery continues to be very successful at relieving pain and restoring joint function, its longevity is challenged by wear and loosening of the implant components. Numerous techniques have been developed to assess polyethylene wear in TKPs during simulator trials and retrieval studies [2] but these are associated with certain limitations. Recently, the use of micro-CT imaging has become widespread, including for wear assessment in retrieved polyethylene liners [10,15,16,27]. In this study, we sought to validate the use of advanced 3D optical scanning techniques to experimentally establish the areas most affected by wear in order to gather basic information to allow a better design of prosthetic knee joints in the future.

Our results clearly show increased wear behavior of the mobile specimens with respect to fixed specimens. The wear mapping results (Figure 5, Figure 6 and Figure 7) are in perfect agreement with the results of the gravimetric tests shown in Figure 4, from a qualitative point of view. With respect to the quantitative analysis of the wear rates, the weighing method provides results slightly different from the new proposed optical method. The deviation among the results is almost 10% in all the cases. We can ascribe this deviation to various causes. First of all, even if the gravimetric results have been obtained by the samples already matted with the powder, an uncertainty of the gravimetric results must be considered due to the uncertainty regarding the quantity of absorbed fluid during the pre-treatment of the prosthesis.

Indirect evaluations of the wear rates by means of relationships between weight and volume, based on the density, have not been taken into account because the evaluation of the worn volumes in the weighing method presumes the knowledge of the exact density of the material, and the material is assumed to be homogenous. From the maps obtained by the fixed menisci analysis, we can conclude that the butterfly alignment method results in a lower wear rate but the wear distribution over the worn surface maintains the same trend. The quantitative results show that the calculation of the wear rate considering the butterfly area, is the correct approach as the results are closer to the gravimetric results.

The 3D scanning method considers the loss of material in terms of volume and the result is not affected by errors due to eventual different liquid absorptions that occurred during the sample preparation and different density distributions. Those elements represent further sources of error in the gravimetric analysis.

Taking all those considerations into account, the 3D scanning method can be considered reliable and more accurate than the gravimetric one, as the only error source ascribable to the measurement in this case derives from the uncertainty of the scanning device. This can be overcome by using metrologically certified devices.

Therefore, to the author’s knowledge, this is the first report on this matter to use a 3D optical scanner to assess the wear of total knee prostheses. In the scientific literature, a few works can be found about the use of a digital procedure in different matters to evaluate 3D wear distribution. For instance, in previous works [21,28], a 3D optical non-contact scanner was used to validate the better efficiency in terms of wear resistance of a new designed industrial component. The advantage of the surface analysis technique described in this report is that it allows quantification of local regions of deviation for the entire total knee prostheses surface, including the articular and backside surfaces. However, the present study has a number of limitations due, mainly, to the number of specimens. In addition, the wear map characterizations were done at the end of wear tests. With respect to the other wear evaluation methods and in particular the use of CMM and weighing methods, the technique proposed in this paper provides many advantages. For example, with respect to the use of CMM, the new proposed optical method permits the roughness and morphology of the specimen to be preserved. With respect to the weighing method, the proposed technique permits the direct evaluation of the worn volumes, also overcoming the uncertainty due to the absorption of fluid during the pre-treatment of the prosthesis as well as allowing the surface distribution of the material loss to be displayed. 

An evolution of this investigation would focus on the wear map of the bearing surface at every wear interval so as to map the wear scar development and thus true pre-wear data would be available for comparison. This would also allow for pre-test component surface digitization using reverse engineering techniques and give a true representation of the component zero-cycle. Finally, a comparison between these in vitro results with explanted knee prostheses could help surgeons. Further tests should be performed on this matter because, to our knowledge, no previous studies have been reported on a similar method, therefore a direct comparison is not yet possible.

## 5. Conclusions

This paper summarized the use of a new approach to better quantify the contact zone on total knee prostheses by using a new digital method to evaluate and assess 3D wear distribution over the surface of TKPs. The results of the present study have been validated, taking into account the qualitative fitting with the gravimetric results. Furthermore, the results have shown that mobile TKPs have lower wear resistance with respect to fixed TKPs.

In conclusion, we have described a technique to create 3D deviation maps for the articular and backside surfaces of menisci polyethylene using a 3D optical scanner. The technique may also be applicable for wear analysis in wear simulator studies of other polyethylene joint replacement components, such as liner or ankle medical devices. Further studies can be done by applying the method presented in this paper in order to assess whether similar wear patterns and wear rates can be observed on retrieved TKPs.

## Figures and Tables

**Table 1:** Technical specifications and performance parameters.

Specs	ScanRider 1.2
Standard Resolution (mm)	<0.05
Precision (Standard Deviation in mm)	<0.03
Average Error (Accuracy in mm) *	<0.01
Working Distance (mm)	120
Number of Triangles for Each Scan	Up to 2.500.000
Output Format	STL
Projector	DLP 600 lm
Camera	B/W 1.3 Megapixel

* For a scanning volume of 60 × 50 × 50 mm.

**Table 2:** Wear rates comparison at the end of the wear test.

Fixed Menisci Nr. *	Mass Wear Rate %	Volumetric Wear Rate %	Deviation %
#2	0.089	**0.099**	**10**
#3	0.052	**0.060**	**13**
#4	0.170	**0.170**	**0**

* Meniscus #1 is the check.
